# Professional Honor

**Published:** 1857-12

**Authors:** 


"*Professional Honor;*—Not that *reflected* honor which our profession has been so free to lavish, even upon the unworthy, but that which has given character and perpetuity to medical science. In this we find the text for a few observations.

"The practitioner of medicine, who has acquired the confidence of community, sustains a relation to society which cannot be secured by any other accomplishment. He is not only entrusted with the life of his fellows, but obtains a passport which freely admits him to the innermost apartments of *home*, where he is even permitted to hold the secrets and share the sympathies of the heart. What bond of security has he given for these chartered rights and privileges? His *honor*, endorsed by the profession as a whole, and this ought to be sufficient. But, alas! poor human nature too often finds in *our* profession melancholy examples of its infirmities. Neither great acquirements, the vows of religion, nor honorable position, always afford undoubted assurance against the violation and forfeiture of the sacred trust. Even carnal lust has invaded the sanctity of home, under the guise of professional honor, prostituting the highest prerogative to the gratification of sensual desires. If we may judge from numerous individual instances, there seems to be a prevalent disposition to lay aside moral character, to ignore the code of medical ethics, to advertise medical talent and acquirement, to loan professional honor for the ends of justice, and even to become a party to crime for a paltry consideration. The editor of the American Medical Monthly tells us in the September number, that 'there would have been no difficulty in obtaining the services of a doctor, who, for a thousand dollars or less, would have done all that Dr. Uhl was asked to do.'

"However reluctantly we may admit the truth of this remark, it is, indeed, a humiliating commentary upon the honor of our profession.

"What has aroused the attention of the medical public to the subject of 'criminal abortion,' urging it upon the attention of the State Medical Societies, calling forth fervent and lengthy editorials from the medical press? What is it, but the facility and readiness with which medical men have permitted themselves to be persuaded from the legitimate path of *professional duty and professional honor?* It has even been said that our statutes, which were designed to recognize and punish this infamous crime, have been rendered null and void by the apparent sanction of respectable medical men.

"Is it not time, then, for even the respectable members of the profession, to consider whether they are not entertaining loose opinions upon this subject, whether their influence towards the suppression of the evil is not at least negative. Popular opinion upon the subject of infanticide at the present time more fitly belongs to an age of barbarism.

"The destruction of fœtal life before the time of quickening, seems not to be regarded as a criminal offence, either in the sight of God or man. Hence the enormous sale of nostrums *that 'must not be taken in the early months of pregnancy.'* Hence, too, the numerous applications and entreaties which every physician has, to violate physiological, civil and moral law, by the sacrifice of embrionic life. These ought to be startling truths, and sufficient to prompt every respectable member of our profession to consider the obligation which he owes to community and his own honor, and to labor with zeal to correct this morbid public sentiment.

"The *character* of the medical profession, in every community, is just what medical men choose to make it. If they are true to its highest interests, and worthy of its mission, they will ever preserve its honor. R."

We copy the above from the Medical Independent; it is one of a number of like articles which we have noticed in the editorial columns of our exchanges, and we earnestly hope that the universal sentiment of the periodical medical press may extend to the daily journals. Upon this point at least, all the conflicting sentiments of the profession must unite, and the monstrous advertisements which we can see in almost every daily paper, of "*Female pills which must not be taken in the early months of pregnancy, as they are sure to produce abortion,*" must be looked upon with horror by *every man.* We do not know whether these villains evade the letter of the law by such a circumlocution, but we hope that the matter may soon be decided by our tribunals, and such men be made an example of. In the mean time we cannot but think that it is the solemn duty of every physician to refuse countenance and patronage to every druggist who lends himself to such black and wholesale iniquity. F.

